# The genome sequence of the acalyptrate fly *Dryomyza anilis* Fallén, 1820

**DOI:** 10.12688/wellcomeopenres.22884.1

**Published:** 2024-08-12

**Authors:** Olga Sivell, Duncan Sivell, Judith A. Webb, Ryan Mitchell, Michael Ashworth, Natural History Museum Genome Acquisition Lab, Darwin Tree of Life Barcoding collective, Wellcome Sanger Institute Tree of Life Management, Samples and Laboratory team, Wellcome Sanger Institute Scientific Operations: Sequencing Operations, Wellcome Sanger Institute Tree of Life Core Informatics team, Tree of Life Core Informatics collective, Darwin Tree of Life Consortium

**Affiliations:** 1 Natural History Museum, London, England, UK; 2 Ecological Consultant, Kidlington, England, UK; 3 Independent researcher, Sligo, County Sligo, Ireland; 4 Independent researcher, Yeovil, England, UK

**Keywords:** Dryomyza anilis, Hooded acalyptrate fly, genome sequence, chromosomal, Diptera

## Abstract

We present a genome assembly from an individual male acalyptrate fly *Dryomyza anilis* (Arthropoda; Insecta; Diptera; Dryomyzidae). The genome sequence has a total length of 656.60 megabases. Most of the assembly is scaffolded into 7 chromosomal pseudomolecules, including the X and Y sex chromosomes. The mitochondrial genome has also been assembled and is 16.48 kilobases in length.

## Species taxonomy

Eukaryota; Opisthokonta; Metazoa; Eumetazoa; Bilateria; Protostomia; Ecdysozoa; Panarthropoda; Arthropoda; Mandibulata; Pancrustacea; Hexapoda; Insecta; Dicondylia; Pterygota; Neoptera; Endopterygota; Diptera; Brachycera; Muscomorpha; Eremoneura; Cyclorrhapha; Schizophora; Acalyptratae; Sciomyzoidea; Dryomyzidae; *Dryomyza*; *Dryomyza anilis* Fallén, 1820 (NCBI:txid169445).

## Background

**Figure 1.**
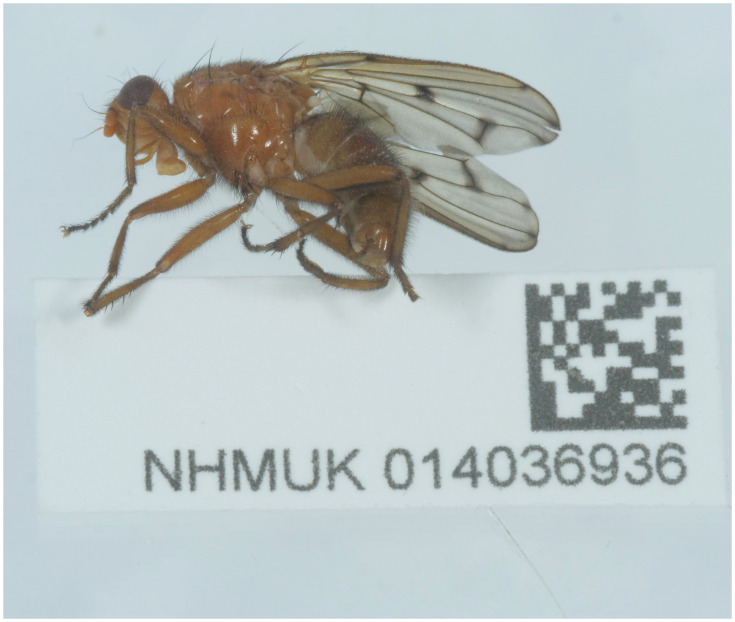
Photograph of *Dryomyza anilis* (idDryAnil1) specimen used for Hi-C sequencing.

*Dryomyza anilis* Fallén, 1820 ( Figure 1) is one of three species from the family Dryomyzidae occurring in Britain, the two others belonging to the genus *Dryope* Robineau-Desvoidy, 1830. *D. anilis* is common and widely distributed across Britain and Ireland. A Holarctic species, it occurs in Europe, Korea, Russia and in North America from Alaska to Labrador, extending south to Oregon, Illinois, and West Virginia ( Evenhuis & Pape, 2024; Mathis & Sueyoshi, 2011). In Britain this species is on the wing from May to November, peaking in June; but is occasionally collected during the winter ( NBN Atlas Partnership, 2024). Before 2011 this species was referred to as *Neuroctena anilis* on the British checklist ( Chandler, 1998) and will appear under that name in some of the literature.

*Dryomyza anilis* is one of the larger British acalyptrates, with a wing length of 5–10 mm ( Falk, 2005). It is yellow, orange or brown in colour, with two dark spots on the wing veins r-m and dm-cu and clouding on the apical parts of veins R _2+3_, R _4+5_ and M. In *D. anilis* vein R _1_ is setose along its whole length, the arista is bare on the apical two thirds (pubescent in *Dryope*), the frons protrudes forward above the antennal bases and the lunule is obscured. The male genitalia are distinct ( Falk, 2005; Mathis & Sueyoshi, 2011; Steyskal, 1957). The egg and terminal segment of the larva were illustrated by Portschinsky (1910) and the mature larva was described and illustrated by Smith (1980). Barnes (1984) provided detailed descriptions and illustrations of all immature stages including the cephalopharyngeal skeleton.

The mating behaviour and fertilisation success in *Dryomyza anilis* were investigated by Otronen ( 1989; 1990; 1993; 1994) and Otronen and Siva-Jothy (1991). Barnes (1984) reported that eggs are laid every 2–10 days, with up to 48 eggs per day. They are usually scattered singly, but occasionally laid side by side in rows of 2 to 5 ( Barnes, 1984). The incubation period is approximately 24 hours. The larvae develop in rotting matter such as carrion, rotting fungi and excrement, however, they are unable to reach maturity in decaying plant matter, e.g. rotting grass, decaying pumpkin flesh, decaying lettuce, or cow manure (Foote in Burger *et al.,* 1980). Burger *et al.* (1980) observed larvae feeding and pupating on a hamburger, dead earthworms, dead crane flies, dead polygyrid snails, a dead milkweed caterpillar, a dead slug, and rotting agaric mushrooms. Barnes (1984) also reared larvae on various crushed insects and on chicken liver. In laboratory conditions reared males lived between 28 to 178 days and females 26 to 167 days ( Barnes, 1984).

The genome of *Dryomyza anilis* was sequenced as part of the Darwin Tree of Life Project, a collaborative effort to sequence all named eukaryotic species in the Atlantic Archipelago of Britain and Ireland. Here we present a chromosomally complete genome sequence for *Dryomyza anilis*, based on one male specimen from Parsonage Moor, Abington, England, UK.

## Genome sequence report

The genome of an adult male *Dryomyza anilis* was sequenced using Pacific Biosciences single-molecule HiFi long reads, generating a total of 31.14 Gb (gigabases) from 2.57 million reads, providing approximately 52-fold coverage. Primary assembly contigs were scaffolded with chromosome conformation Hi-C data, which produced 115.43 Gbp from 764.41 million reads, yielding an approximate coverage of 176-fold. Specimen and sequencing information is summarised in Table 1.

**Table 1.** Specimen and sequencing data for *Dryomyza anilis*.

Project information			
**Study title**	*Dryomyza anilis*		
**Umbrella BioProject**	PRJEB60311		
**Species**	*Dryomyza anilis*		
**BioSample**	SAMEA110049601		
**NCBI taxonomy ID**	169445		
Specimen information			
**Technology**	**ToLID**	**BioSample accession**	**Organism part**
**PacBio long read sequencing**	idDryAnil2	SAMEA14448859	head and thorax
**Hi-C sequencing**	idDryAnil1	SAMEA11025371	head and thorax
Sequencing information			
**Platform**	**Run accession**	**Read count**	**Base count (Gb)**
**Hi-C Illumina NovaSeq 6000**	ERR10968292	7.64e+08	115.43
**PacBio Sequel IIe**	ERR10962209	2.57e+06	31.14

**Figure 2.**
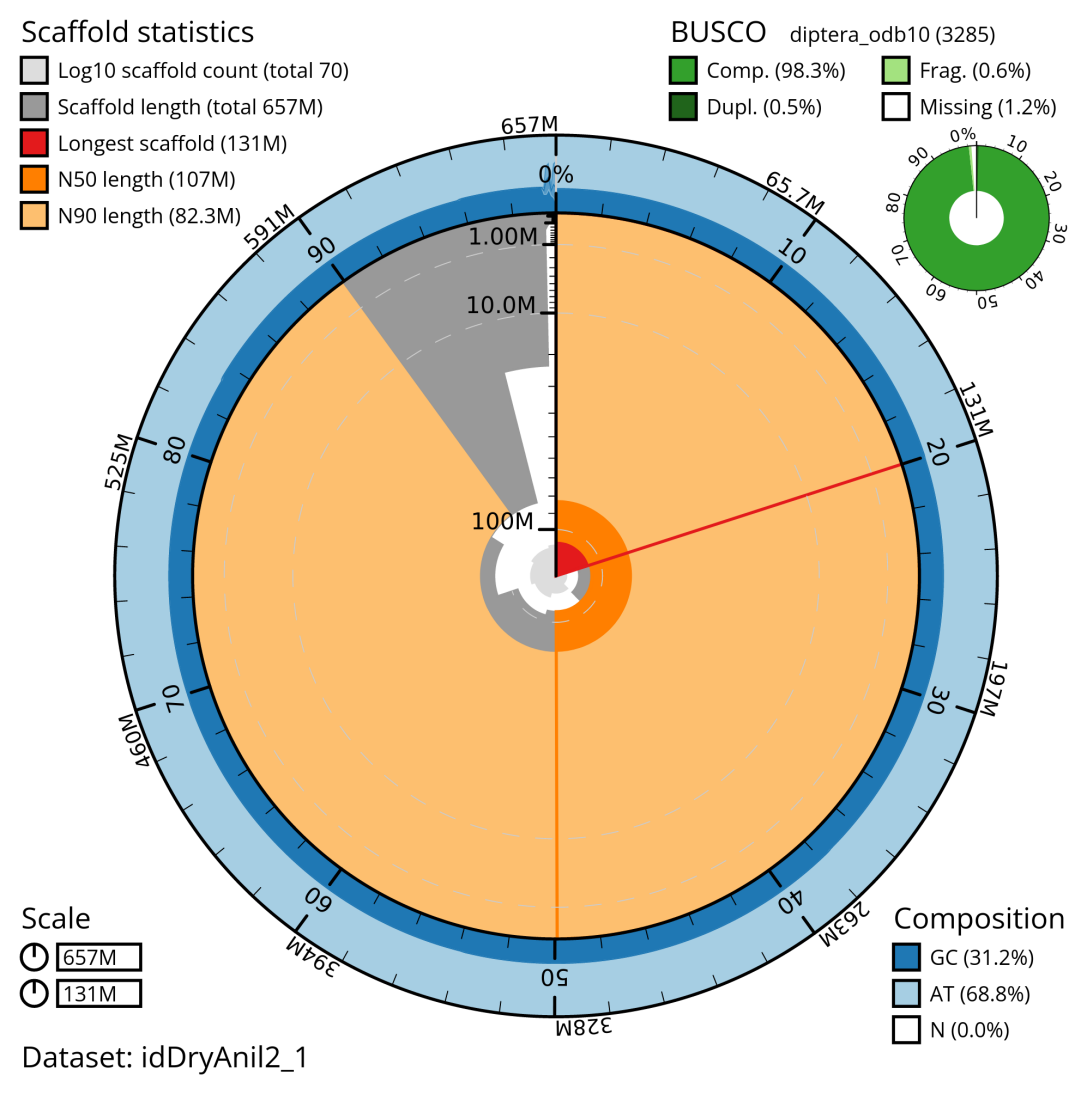
Genome assembly of *Dryomyza anilis*, idDryAnil2.1: metrics. The BlobToolKit snail plot shows N50 metrics and BUSCO gene completeness. The main plot is divided into 1,000 size-ordered bins around the circumference with each bin representing 0.1% of the 656,620,501 bp assembly. The distribution of scaffold lengths is shown in dark grey with the plot radius scaled to the longest scaffold present in the assembly (131,452,544 bp, shown in red). Orange and pale-orange arcs show the N50 and N90 scaffold lengths (106,770,135 and 82,254,885 bp), respectively. The pale grey spiral shows the cumulative scaffold count on a log scale with white scale lines showing successive orders of magnitude. The blue and pale-blue area around the outside of the plot shows the distribution of GC, AT and N percentages in the same bins as the inner plot. A summary of complete, fragmented, duplicated and missing BUSCO genes in the diptera_odb10 set is shown in the top right. An interactive version of this figure is available at https://blobtoolkit.genomehubs.org/view/idDryAnil2_1/dataset/idDryAnil2_1/snail.

**Figure 3.**
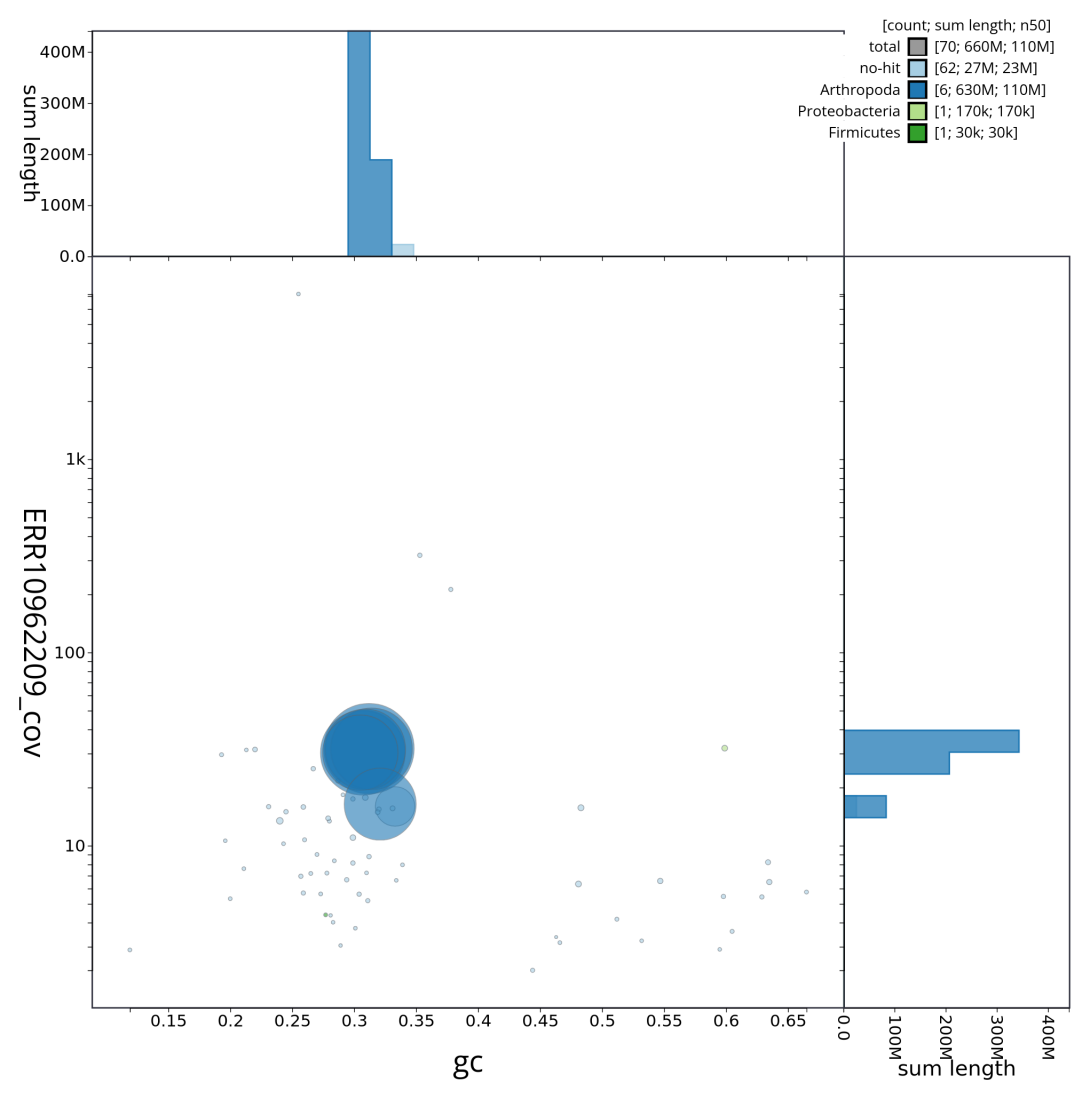
Genome assembly of *Dryomyza anilis*, idDryAnil2.1: Blob plot of base coverage in ERR10962209 against GC proportion for sequences in assembly idDryAnil2.1. Sequences are coloured by phylum. Circles are sized in proportion to sequence length. Histograms show the distribution of sequence length sum along each axis. An interactive version of this figure is available at https://blobtoolkit.genomehubs.org/view/idDryAnil2_1/dataset/idDryAnil2_1/blob.

**Figure 4.**
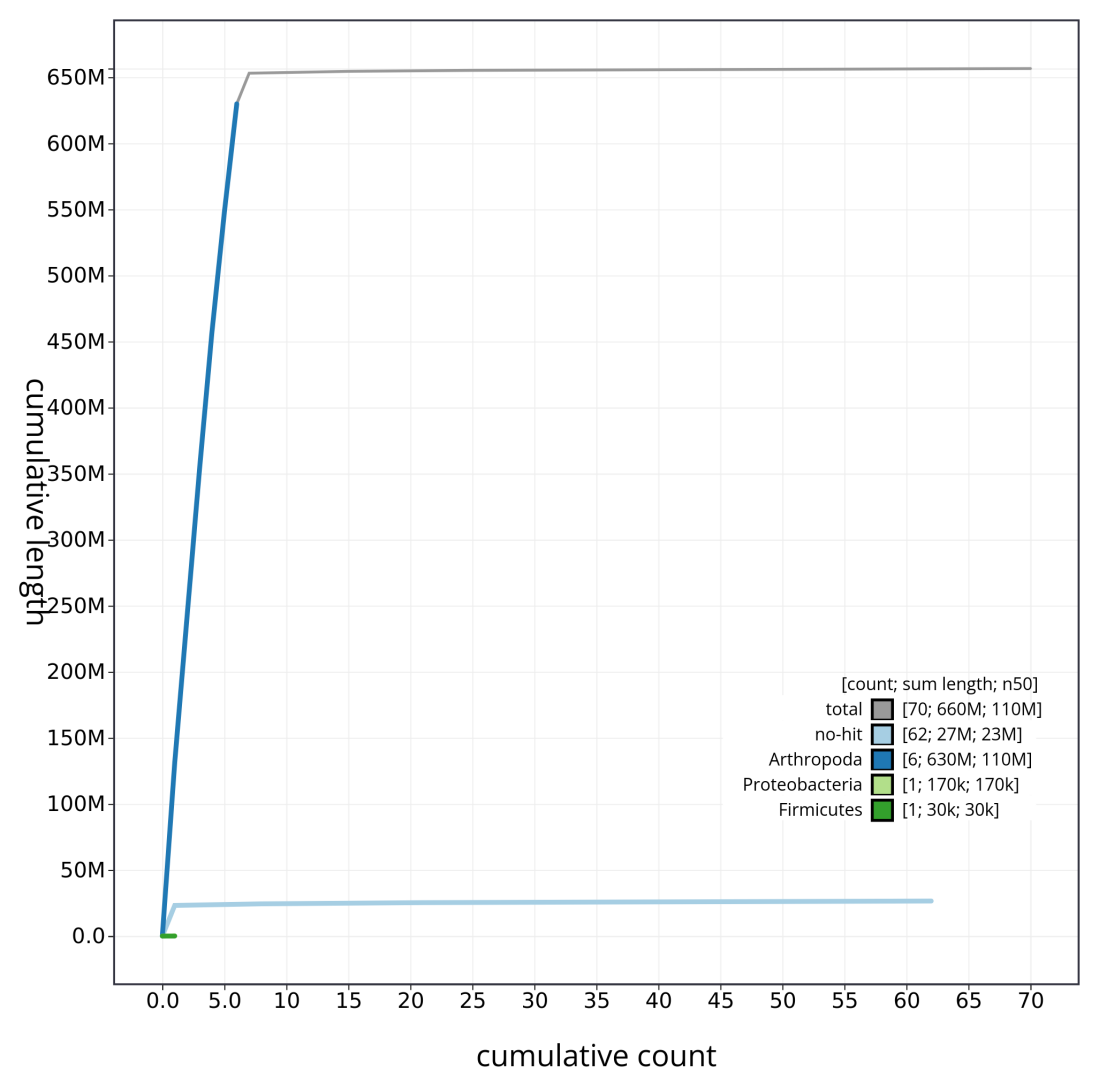
Genome assembly of *Dryomyza anilis* idDryAnil2.1: BlobToolKit cumulative sequence plot. The grey line shows cumulative length for all sequences. Coloured lines show cumulative lengths of sequences assigned to each phylum using the buscogenes taxrule. An interactive version of this figure is available at https://blobtoolkit.genomehubs.org/view/idDryAnil2_1/dataset/idDryAnil2_1/cumulative.

**Figure 5.**
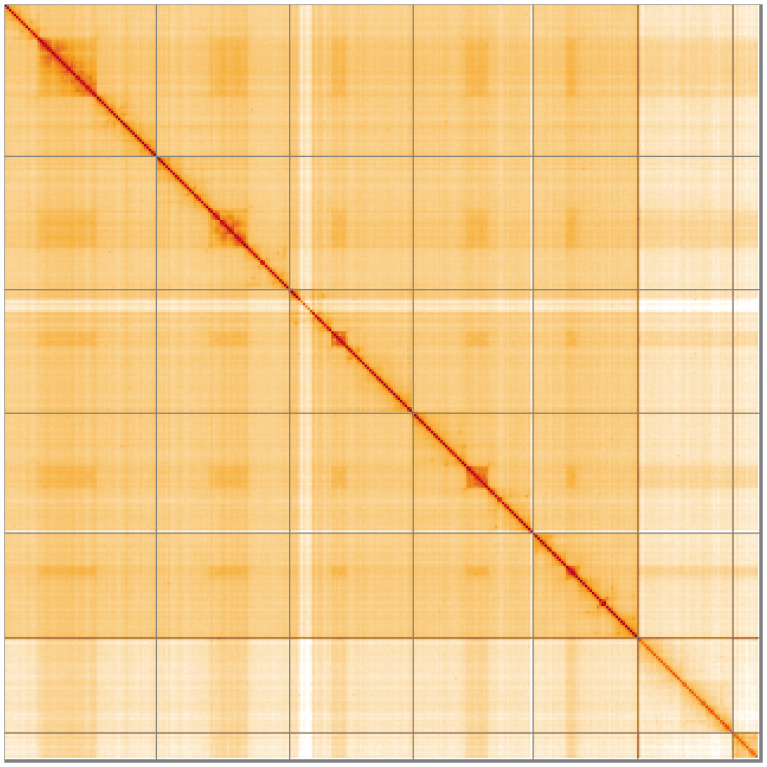
Genome assembly of *Dryomyza anilis* idDryAnil2.1: Hi-C contact map of the idDryAnil2.1 assembly, visualised using HiGlass. Chromosomes are shown in order of size from left to right and top to bottom. An interactive version of this figure may be viewed at https://genome-note-higlass.tol.sanger.ac.uk/l/?d=TK6XO1QuT9-ZWJPQMIT_4w.

Manual assembly curation corrected 49 missing joins or mis-joins and two haplotypic duplications, reducing the scaffold number by 20.45%. The final assembly has a total length of 656.60 Mb in 69 sequence scaffolds with a scaffold N50 of 106.8 Mb ( Table 2), with 422 gaps. The snail plot in Figure 2 provides a summary of the assembly statistics, while the distribution of assembly scaffolds on GC proportion and coverage is shown in Figure 3. The cumulative assembly plot in Figure 4 shows curves for subsets of scaffolds assigned to different phyla. Most (99.46%) of the assembly sequence was assigned to 7 chromosomal-level scaffolds, representing 5 autosomes and the X and Y sex chromosomes. Chromosome-scale scaffolds confirmed by the Hi-C data are named in order of size ( Figure 5; Table 3). Sex chromosome annotation was guided by synteny alignments to *Coelopa pilipes* (GCA_947389925.1) ( Butlin *et al.*, 2024). While not fully phased, the assembly deposited is of one haplotype. Contigs corresponding to the second haplotype have also been deposited. The mitochondrial genome was also assembled and can be found as a contig within the multifasta file of the genome submission.

**Table 2.** Genome assembly data for *Dryomyza anilis*, idDryAnil2.1.

Genome assembly		
Assembly name	idDryAnil2.1	
Assembly accession	GCA_951804985.1	
*Accession of alternate haplotype*	*GCA_951804995.1*	
Span (Mb)	656.60	
Number of contigs	492	
Contig N50 length (Mb)	3.2	
Number of scaffolds	69	
Scaffold N50 length (Mb)	106.8	
Longest scaffold (Mb)	131.45	
Assembly metrics *		*Benchmark*
Consensus quality (QV)	59.3	*≥ 50*
*k*-mer completeness	100.0%	*≥ 95%*
BUSCO **	C:98.3%[S:97.8%,D:0.5%], F:0.6%,M:1.2%,n:3,285	*C ≥ 95%*
Percentage of assembly mapped to chromosomes	99.46%	*≥ 95%*
Sex chromosomes	XY	*localised homologous pairs*
Organelles	Mitochondrial genome: 16.48 kb	*complete single alleles*

* Assembly metric benchmarks are adapted from column VGP-2020 of “Table 1: Proposed standards and metrics for defining genome assembly quality” from Rhie *et al.* (2021). ** BUSCO scores based on the diptera_odb10 BUSCO set using version 5.3.2. C = complete [S = single copy, D = duplicated], F = fragmented, M = missing, n = number of orthologues in comparison. A full set of BUSCO scores is available at https://blobtoolkit.genomehubs.org/view/idDryAnil2_1/dataset/idDryAnil2_1/busco.

**Table 3.** Chromosomal pseudomolecules in the genome assembly of *Dryomyza anilis*, idDryAnil2.

INSDC accession	Name	Length (Mb)	GC%
OX638130.1	1	131.45	31.0
OX638131.1	2	115.26	30.5
OX638132.1	3	106.77	31.5
OX638133.1	4	103.6	31.0
OX638134.1	5	90.55	30.5
OX638135.1	X	82.25	32.0
OX638136.1	Y	23.2	33.5
OX638137.1	MT	0.02	25.5

The estimated Quality Value (QV) of the final assembly is 59.3 with *k*-mer completeness of 100.0%, and the assembly has a BUSCO v5.3.2 completeness of 98.3% (single = 97.8%, duplicated = 0.5%), using the diptera_odb10 reference set ( *n* = 3,285).

Metadata for specimens, BOLD barcode results, spectra estimates, sequencing runs, contaminants and pre-curation assembly statistics are given at https://links.tol.sanger.ac.uk/species/169445.

## Methods

### Sample acquisition

An adult male *Dryomyza anilis* (specimen ID NHMUK014036859, ToLID idDryAnil2) was collected from Parsonage Moor, Abington, England, UK (latitude 51.69, longitude –1.33) on 2021-06-19 using an aerial net. The specimen was collected by Duncan Sivell, Olga Sivell, Ryan Mitchell and Judy Webb and identified by Ryan Mitchell. The specimen used for Hi-C sequencing (specimen ID NHMUK014036936, ToLID idDryAnil1) was an adult specimen collected from Kenall Vale, Truro, England, UK (latitude 50.2, longitude –5.15) on 2021-06-30 using an aerial net. This specimen was collected and identified by Mike Ashworth (independent researcher). Figure 1 is a photograph of the latter specimen.

The initial species identification was verified by an additional DNA barcoding process according to the framework developed by Twyford *et al*. (2024). Small samples were dissected from the specimens and stored in ethanol, while the remaining parts of the specimens were shipped on dry ice to the Wellcome Sanger Institute (WSI). The tissue was lysed, the COI marker region was amplified by PCR, and amplicons were sequenced and compared to the BOLD database, confirming the species identification ( Crowley *et al.*, 2023). Following whole genome sequence generation, the relevant DNA barcode region was also used alongside the initial barcoding data for sample tracking at the WSI ( Twyford *et al.*, 2024). The standard operating procedures for Darwin Tree of Life barcoding have been deposited on protocols.io ( Beasley *et al.*, 2023).

### Nucleic acid extraction

The workflow for high molecular weight (HMW) DNA extraction at the WSI Tree of Life Core Laboratory includes a sequence of core procedures: sample preparation and homogenisation, DNA extraction, fragmentation, and purification. Detailed methods are publicly available on protocols.io ( Denton *et al.*, 2023b).

The idDryAnil2 sample was weighed and dissected on dry ice ( Jay *et al.*, 2023) and tissue from the head and thorax was homogenised using a PowerMasher II tissue disruptor ( Denton *et al.*, 2023a). HMW DNA was extracted using the Automated MagAttract v1 protocol ( Sheerin *et al.*, 2023). DNA was sheared into an average fragment size of 12–20 kb in a Megaruptor 3 system with speed setting 30 ( Todorovic *et al.*, 2023). Sheared DNA was purified by solid-phase reversible immobilisation, using AMPure PB beads to eliminate shorter fragments and concentrate the DNA ( Strickland *et al.*, 2023). The concentration of the sheared and purified DNA was assessed using a Nanodrop spectrophotometer and Qubit Fluorometer using the Qubit dsDNA High Sensitivity Assay kit. Fragment size distribution was evaluated by running the sample on the FemtoPulse system.

### Sequencing

Pacific Biosciences HiFi circular consensus DNA sequencing libraries were constructed according to the manufacturers’ instructions. DNA sequencing was performed by the Scientific Operations core at the WSI on a Pacific Biosciences Sequel IIe instrument. Hi-C data were also generated from the head and thorax tissue of idDryAnil1 using the Arima-HiC v2 kit. The Hi-C sequencing was performed using paired-end sequencing with a read length of 150 bp on the Illumina NovaSeq 6000 instrument.

### Genome assembly, curation and evaluation

***Assembly***

Original assembly of HiFi reads is performed using Hifiasm ( Cheng *et al.*, 2021) with the --primary option. Haplotypic duplications were identified and removed using purge_dups ( Guan *et al.*, 2020). Hi-C reads were mapped to the primary contigs using bwa-mem2 ( Vasimuddin *et al.*, 2019), and these contigs were scaffolded using the provided Hi-C data ( Rao *et al.*, 2014) in YaHS ( Zhou *et al.*, 2023) using the --break option. Scaffolded assemblies were evaluated using Gfastats ( Formenti *et al.*, 2022), BUSCO ( Manni *et al.*, 2021) and MERQURY.FK ( Rhie *et al.*, 2020).

The mitochondrial genome was assembled using MitoHiFi ( Uliano-Silva *et al.*, 2023), which runs MitoFinder ( Allio *et al.*, 2020) and uses these annotations to select the final mitochondrial contig and to ensure the general quality of the sequence.

***Assembly curation***

The assembly was decontaminated using the Assembly Screen for Cobionts and Contaminants (ASCC) pipeline (article in preparation). Manual curation was primarily conducted using PretextView ( Harry, 2022), with additional insights provided by JBrowse2 ( Diesh *et al.*, 2023) and HiGlass ( Kerpedjiev *et al.*, 2018). Scaffolds were visually inspected and corrected as described by Howe *et al*. (2021). Any identified contamination, missed joins, and mis-joins were corrected, and duplicate sequences were tagged and removed. Sex chromosomes were identified by synteny. The entire process is documented at https://gitlab.com/wtsi-grit/rapid-curation (article in preparation).

***Evaluation of the final assembly***

A Hi-C map for the final assembly was produced using bwa-mem2 ( Vasimuddin *et al.*, 2019) in the Cooler file format ( Abdennur & Mirny, 2020). To assess the assembly metrics, the *k*-mer completeness and QV consensus quality values were calculated in Merqury ( Rhie *et al.*, 2020). This work was done using Nextflow ( Di Tommaso *et al.*, 2017) DSL2 pipelines “sanger-tol/readmapping” ( Surana *et al.*, 2023a) and “sanger-tol/genomenote” ( Surana *et al.*, 2023b). The genome was analysed within the BlobToolKit environment ( Challis *et al.*, 2020) and BUSCO scores ( Manni *et al.*, 2021; Simão *et al.*, 2015) were calculated.

The genome evaluation pipelines were developed using the nf-core tooling ( Ewels *et al.*, 2020), use MultiQC ( Ewels *et al.*, 2016), and make extensive use of the Conda package manager, the Bioconda initiative ( Grüning *et al.*, 2018), the Biocontainers infrastructure ( da Veiga Leprevost *et al.*, 2017), and the Docker ( Merkel, 2014) and Singularity ( Kurtzer *et al.*, 2017) containerisation solutions.

Table 4 contains a list of relevant software tool versions and sources.

**Table 4.** Software tools: versions and sources.

Software tool	Version	Source
BlobToolKit	4.2.1	https://github.com/blobtoolkit/blobtoolkit
BUSCO	5.3.2	https://gitlab.com/ezlab/busco
bwa-mem2	2.2.1	https://github.com/bwa-mem2/bwa-mem2
Gfastats	1.3.6	https://github.com/vgl-hub/gfastats
Hifiasm	0.16.1-r375	https://github.com/chhylp123/hifiasm
HiGlass	1.11.6	https://github.com/higlass/higlass
Merqury	MerquryFK	https://github.com/thegenemyers/MERQURY.FK
MitoHiFi	2	https://github.com/marcelauliano/MitoHiFi
PretextView	0.2	https://github.com/wtsi-hpag/PretextView
purge_dups	1.2.3	https://github.com/dfguan/purge_dups
sanger-tol/ascc	-	https://github.com/sanger-tol/ascc
sanger-tol/genomenote	v1.0	https://github.com/sanger-tol/genomenote
sanger-tol/readmapping	1.1.0	https://github.com/sanger-tol/readmapping/tree/1.1.0
YaHS	yahs-1.1.91eebc2	https://github.com/c-zhou/yahs

### Wellcome Sanger Institute – Legal and Governance

The materials that have contributed to this genome note have been supplied by a Darwin Tree of Life Partner. The submission of materials by a Darwin Tree of Life Partner is subject to the **‘Darwin Tree of Life Project Sampling Code of Practice’**, which can be found in full on the Darwin Tree of Life website here. By agreeing with and signing up to the Sampling Code of Practice, the Darwin Tree of Life Partner agrees they will meet the legal and ethical requirements and standards set out within this document in respect of all samples acquired for, and supplied to, the Darwin Tree of Life Project. 

Further, the Wellcome Sanger Institute employs a process whereby due diligence is carried out proportionate to the nature of the materials themselves, and the circumstances under which they have been/are to be collected and provided for use. The purpose of this is to address and mitigate any potential legal and/or ethical implications of receipt and use of the materials as part of the research project, and to ensure that in doing so we align with best practice wherever possible. The overarching areas of consideration are:

•   Ethical review of provenance and sourcing of the material

•   Legality of collection, transfer and use (national and international) 

Each transfer of samples is further undertaken according to a Research Collaboration Agreement or Material Transfer Agreement entered into by the Darwin Tree of Life Partner, Genome Research Limited (operating as the Wellcome Sanger Institute), and in some circumstances other Darwin Tree of Life collaborators.

## Data Availability

European Nucleotide Archive: *Dryomyza anilis*. Accession number PRJEB60311; https://identifiers.org/ena.embl/PRJEB60311 ( Wellcome Sanger Institute, 2023). The genome sequence is released openly for reuse. The *Dryomyza anilis* genome sequencing initiative is part of the Darwin Tree of Life (DToL) project. All raw sequence data and the assembly have been deposited in INSDC databases. The genome will be annotated using available RNA-Seq data and presented through the Ensembl pipeline at the European Bioinformatics Institute. Raw data and assembly accession identifiers are reported in Table 1 and Table 2.

## Author information

Members of the Natural History Museum Genome Acquisition Lab are listed here: https://doi.org/10.5281/zenodo.12159242.

Members of the Darwin Tree of Life Barcoding collective are listed here: https://doi.org/10.5281/zenodo.12158331

Members of the Wellcome Sanger Institute Tree of Life Management, Samples and Laboratory team are listed here: https://doi.org/10.5281/zenodo.12162482.

Members of Wellcome Sanger Institute Scientific Operations: Sequencing Operations are listed here: https://doi.org/10.5281/zenodo.12165051.

Members of the Wellcome Sanger Institute Tree of Life Core Informatics team are listed here: https://doi.org/10.5281/zenodo.12160324.

Members of the Tree of Life Core Informatics collective are listed here: https://doi.org/10.5281/zenodo.5013541.

Members of the Darwin Tree of Life Consortium are listed here: https://doi.org/10.5281/zenodo.4783558.
