# Supplementary material for: The genome sequence of the acalyptrate fly Dryomyza anilis Fallén, 1820
Source: Wellcome Open Res. 2024 Aug 12;9:440. [Version 1] doi: 10.12688/wellcomeopenres.22884.1 (PMC11472010; doi:10.12688/wellcomeopenres.22884.1)
Supplement: Peer review history

# Reviewer report: Reviewer response for version 1

**DOI:** 10.21956/wellcomeopenres.25199.r99670

**Published:** 2024-10-13

**Authors:** Wenchao Guo

**Affiliations:** 1 Xinjiang Academy of Agricultural Sciences, Urumqi, China

This work presented a genome assembly from an individual male acalyptrate fly *Dryomyza anilis*. The genome sequence had a total length of 656.60 megabases. The assembly is scaffolded in 69 sequence scaffolds, and a scaffold N50 of 106.8 Mb into 7 chromosomal pseudomolecules, including the X and Y sex chromosomes. The mitochondrial genome has also been assembled and is 16.48 kilobases in length. Above all, the procedures including sample acquisition, DNA extraction, sequencing, assembly and evaluation of the final assembly was clearly expressed. However, there's some suggestions to improve this manuscript.

 1. Genome sequence report: Add "The" before Primary assembly contigs were scaffolded.

 2. Add some information about the reason why to genome sequence of this species.

Are sufficient details of methods and materials provided to allow replication by others?

Yes

Is the rationale for creating the dataset(s) clearly described?

Yes

Are the datasets clearly presented in a useable and accessible format?

Yes

Are the protocols appropriate and is the work technically sound?

Yes

Reviewer Expertise:

Agricultural Entomology and Pest Control

I confirm that I have read this submission and believe that I have an appropriate level of expertise to confirm that it is of an acceptable scientific standard.

# Reviewer report: Reviewer response for version 1

**DOI:** 10.21956/wellcomeopenres.25199.r100675

**Published:** 2024-09-30

**Authors:** Mireille Steck

**Affiliations:** 1 University of Hawaiʻi at Mānoa, Honolulu, USA

This is a great genome contribution, it is clear the authors took the time to curate and assemble this data well. As someone unfamiliar with insect morphology, the abbreviations for wing veins were difficult to follow, and little description on how the male genitalia are distinct was provided. It would be nice to see the morphology of the animal summarized a little more.

 Overall the information is well presented and the methods are well documented, I would recommend this to be indexed with little to no revision.

Are sufficient details of methods and materials provided to allow replication by others?

Yes

Is the rationale for creating the dataset(s) clearly described?

Yes

Are the datasets clearly presented in a useable and accessible format?

Yes

Are the protocols appropriate and is the work technically sound?

Yes

Reviewer Expertise:

Invertebrate molecular biology

I confirm that I have read this submission and believe that I have an appropriate level of expertise to confirm that it is of an acceptable scientific standard.
